# Micro/nanorobots for gastrointestinal tract

**DOI:** 10.3389/fchem.2024.1423696

**Published:** 2024-11-08

**Authors:** Ziqi Sui, Chugen Wan, Hefei Cheng, Bin Yang

**Affiliations:** ^1^ Department of Gastroenterology, The Second Affiliated Hospital of Zhejiang Chinese Medical University, Hangzhou, Zhejiang, China; ^2^ Department of Gastroenterology, The First People’s Hospital of Linping District, Hangzhou, Zhejiang, China; ^3^ Department of Gastroenterology, Second Affiliated Hospital of Zhejiang University School of Medicine, Hangzhou, Zhejiang, China

**Keywords:** micro/nanomotors, gastrointestinal tract, targeted delivery, physiological barriers, applications

## Abstract

The application of micro/nanomotors (MNMs) in the gastrointestinal tract has become a Frontier in the treatment of gastrointestinal diseases. These miniature robots can enter the gastrointestinal tract through oral administration, achieving precise drug delivery and therapy. They can traverse mucosal layers and tissue barriers, directly targeting tumors or other lesion sites, thereby enhancing the bioavailability and therapeutic effects of drugs. Through the application of nanotechnology, these MNMs are able to accomplish targeted medication release, regulating drug release in response to either external stimuli or the local biological milieu. This results in reduced side effects and increased therapeutic efficacy. This review summarizes the primary classifications and power sources of current MNMs, as well as their applications in the gastrointestinal tract, providing inspiration and direction for the treatment of gastrointestinal diseases with MNMs.

## 1 Introduction

Recent advances in robotic systems have enabled improvement of micro- and nanoscale robots in medical applications (Abdelmohsen et al., 2014; Sánchez et al., 2015; Zhao et al., 2022). Besides micro/nanorobots for precision surgery, sensing and detoxification, the application in targeted delivery is a promising field in gastrointestinal (GI) tract (Li et al., 2017a). To achieve systemic circulation following oral administration, drugs must undergo a series of steps. These include traversal through the GI tract, interaction with and penetration of the mucus layer, transportation across the epithelial barrier, access to the hepatic portal vein, and eventual entry into the systemic circulation (Zhang et al., 2013). Oral administration is the most common and preferred route due to its non-invasive nature, patient compliance, and ease of administration. However, the complex and unique physicochemical properties of the gastrointestinal tract pose challenges for drug delivery, affecting the absorption of drug molecules. These challenges include poor drug stability, low bioavailability, and limited permeability across membrane barriers (Alqahtani et al., 2021). It has been reported that almost 70% of new chemical entities are eliminated during pre-clinical development because of inadequate oral bioavailability (Gao et al., 2013). Selectively locating therapeutic or imaging agents to specific segments of the GI tract through micro/nanorobots is of considerable prospect (Traverso and Langer, 2015). Because of passive mass transport limitation, existing delivery micro/nanocarriers rely on systemic circulation and lack the force and navigation required for local delivery and tissue penetration (Li et al., 2017a). Microrobots, with their ability to navigate through various gastrointestinal barriers, strong controllable motion, and capability for active drug transport, show promising potential in improving oral drug delivery (Ren et al., 2024).

Hampered by the body’s natural physiological and structural barriers, it still remains an unmet need to develop a biocompatible nano/micro-scale device that can selectively position in a specific segment of the GI tract and actively penetrate into the tissue for prolonged retention (Li et al., 2016). Gastric acid, mucus, and a strictly controlled intestinal epithelium are examples of physiological barriers that limit the bioavailability of drugs (Date et al., 2016). Following oral administration, nanoparticles typically swiftly traverse the oral cavity and esophagus before entering the stomach. Key to this process is the stomach, a hollow and contractile organ that produces gastric juices for digesting (Andretto et al., 2021). However, the presence of gastric acid imposes constraints on the absorption of oral nanomedicines. Consequently, assessing the impact of gastric acid becomes a crucial test for oral nanocarriers, directly linked to the stability of nanocarriers and the therapeutic efficacy of drugs (Zhang S. et al., 2023). The mucus layer in the GI tract serves as a significant obstacle for the absorption of oral nanomedicines. Comprising primarily of water (around 95%), mucins (glycoproteins, approximately 2%–5% w/v), lipids, DNA, antibodies, and cellular debris, this viscoelastic gel layer poses a challenge to the effective uptake of nanomedicines (Boegh and Nielsen, 2015). The protective properties of mucus create challenges for the efficient transit of nanodrugs. Due to their autonomous motion capability, micro/nanorobots can quickly traverse the mucus layer and become internalized by epithelial cells (Wang et al., 2022). Beneath the mucus layer lies the epithelial barrier, which consists of epithelial cells responsible for controlling the absorption of substances from the GI tract into the body or lymphatic circulation (Yang et al., 2019). The self-propulsion of micro/nanorobots provide benefits include better tissue accumulation, faster lysosomal escape, deeper tissue penetration, and increased cellular uptake—all of which contribute to enhanced cellular transcytosis (Liu T. et al., 2022). As a result, the creation of effective drug delivery systems that can surmount these obstacles is essential for enhancing the effectiveness of oral drug delivery. Overcoming these physiological barriers in oral administration could greatly enhance drug delivery efficiency and, consequently, overall efficacy (Zhang S. et al., 2023).

Micro/nanomotors (MNMs) are micro/nanorobots capable of converting diverse energy sources into locomotion or actuation (Soto et al., 2020). MNMs enhance biosensing sensitivity and facilitate efficient drug delivery by leveraging their exceptional attributes, which include enhancing agitation, rapid transportation, active targeting, and deep tissue penetration (Gao et al., 2021). In recent years, various MNMs capable of self-propulsion in fluid have emerged (Fernández Medina et al., 2020). MNMs technology requires a significant amount of power to drive the robots, control their movement, and steer them along predetermined pathways in order to accomplish effective drug delivery. The propulsion mechanism of these MNMs is contingent upon their structural design and chemical composition (Wang and Zhou, 2021). In essence, micromotors have the capability to transform various forms of energy they receive (chemical, magnetic, electric, ultrasound, thermal, light) into motion (Tang et al., 2021; Ji et al., 2021; Zhang et al., 2023b). The actuation of MNMs can be either continuous or discrete. But for the most part it is continuous. For chemically propelled MNMs, It is driven by chemical reactions, and because of the nature of chemical reactions, the drive is usually continuous. For magnetically driven MNMs, it depends on the nature of the driving signal. When the external magnetic field driving signal is continuously varying, the driving of the MNMs will also be continuous. For example, in a magnetic field-driven system, if the direction and strength of the magnetic field change continuously over time, then the MNMs will move along a continuously changing trajectory. When the external magnetic field driving signal is discrete, such as the pulsed magnetic field, the driving of the micro nanosheet will also be discrete. Micro/nanomotors (MNMs) are micro/nanorobots capable of converting diverse energy sources into locomotion or actuation (Soto et al., 2020). MNMs enhance biosensing sensitivity and facilitate efficient drug delivery by leveraging their exceptional attributes, which include enhancing agitation, rapid transportation, active targeting, and deep tissue penetration (Gao et al., 2021). In recent years, various MNMs capable of self-propulsion in fluid have emerged (Fernández Medina et al., 2020). MNMs technology requires a significant amount of power to drive the robots, control their movement, and steer them along predetermined pathways in order to accomplish effective drug delivery. The propulsion mechanism of these MNMs is contingent upon their structural design and chemical composition (Wang and Zhou, 2021). In essence, micromotors have the capability to transform various forms of energy they receive (chemical, magnetic, electric, ultrasound, thermal, light) into motion (Tang et al., 2021; Ji et al., 2021; Zhang et al., 2023b).

**FIGURE 1:**
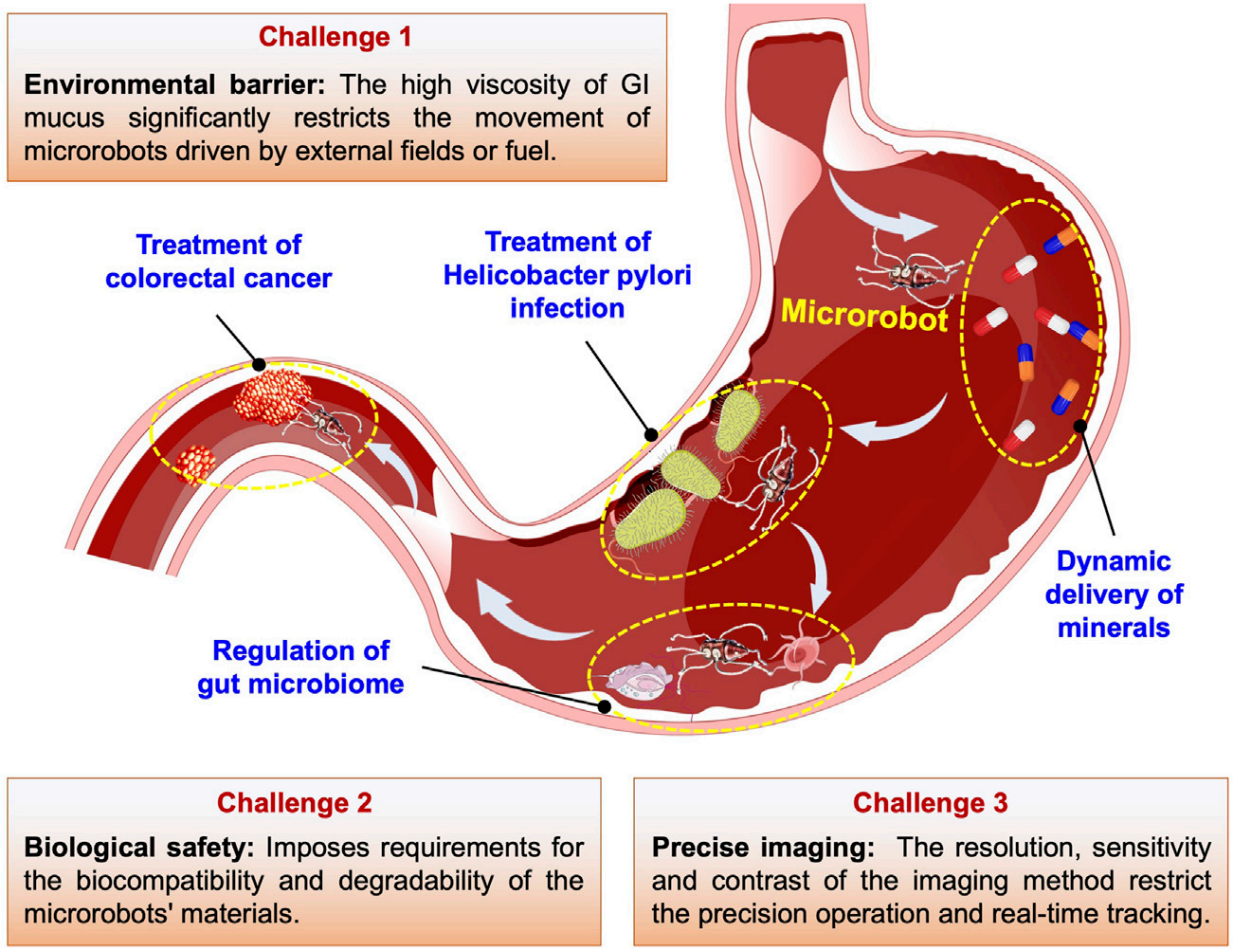
Applications and challenges of MNMs in GI tract.

There are three primary categories of micromotors: chemically propelled micromotors, externally driven micromotors, and biologically powered micromotors. Each category possesses unique characteristics to address the physiological challenges within the GI tract (Zhang S. et al., 2023). The prompt traversal of physiological barriers by micromotors serves a dual purpose: it helps evade metabolism by diverse physiological conditions while enhancing drug delivery efficiency at GI disease sites, reducing non-specific interactions with healthy tissues (Zhou et al., 2019a; Zhang et al., 2024). MNMs can carry drugs through the digestive system to achieve dynamic drug delivery to target areas in the GI tract, which is of great value in terms of both enrichment of the drug in the diseased area and therapeutic effect. In addition, as shown in Figure 1, MNMs have shown potential for the treatment of bacterial infections, microbiota disorders, and tumor lesions in the GI tract. Of course, there are still many challenges for the implementation of MNMs in the treatment of GI disease in clinical practice. In this review, we have summarized the primary classifications and sources of propulsion for MNMs, as well as the latest advancements in their applications in the GI tract. Based on the basic understanding and summary of classifications and propulsion mechanisms of MNMs, we survey the latest research on miniature robots for the targeted delivery and therapy in the GI tract and describe tentative future research trends and important challenge for future research (Figure 1). This review aims to inspire and guide the use of MNMs in the treatment of GI diseases, advancing their practical application in oral drug delivery.

## 2 Classification based on constituent materials

MNMs, which operate at the micron or nanometer scale, are robotic systems characterized by specific chemical compositions and physical properties that enable them to perform precise tasks (Zhang S. et al., 2022). The functions and applications of these robots are determined by their unique chemical compositions and physical properties (Li et al., 2023). MNMs can be categorized as hydrogel-based, exogenously driveable, and bio-based, depending on their inherent characteristics and chemical composition (Soto et al., 2020).

**FIGURE 2:**
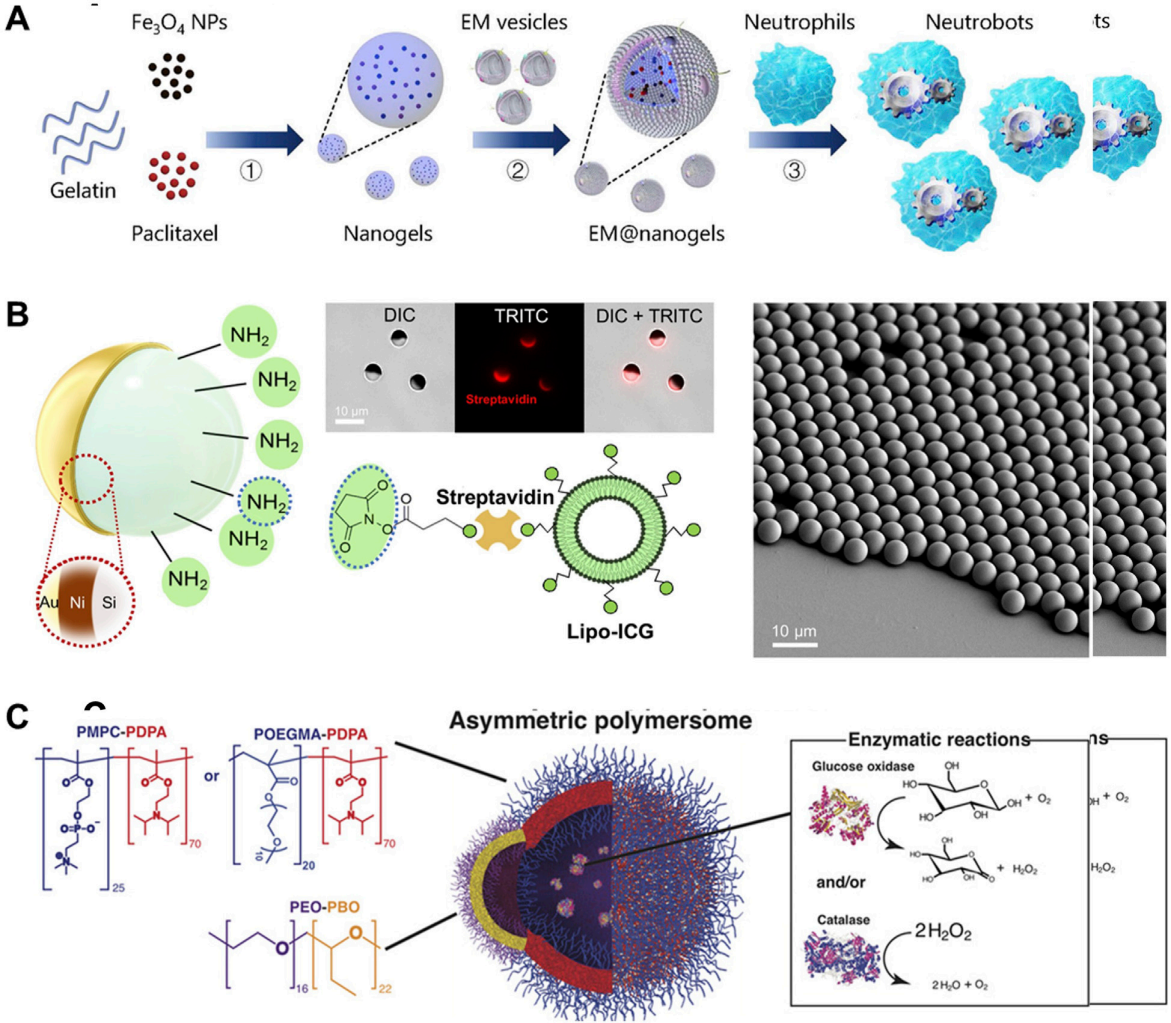
Preparation mechanism of different types of MNMs. **(A)** Preparation process hydrogel-type biohybrid neutrobots (Zhang H. et al., 2021). **(B)** Schematics depicting the coating composition of the microcapillary-sized magnetic microrobots (Wrede et al., 2022). **(C)** The polymersomes encapsulate glucose oxidase and/or catalase enzymes (Joseph et al., 2017). **(A)** Copyright ^©^ 2021, The American Association for the Advancement of Science **(B)** Copyright ^©^ 2022, The American Association for the Advancement of Science **(C)** Copyright ^©^ 2017, The American Association for the Advancement of Science.

(1) Hydrogel-type MNMs, comprising soft and deformable hydrogel materials, exhibit water dispersibility and biocompatibility (Nasution et al., 2022; Wang et al., 2021a). These characteristics render them promising for diverse medical applications, including drug delivery and tissue repair (Wang et al., 2023). Hydrogel type MNMs obtain the target configuration of liquid hydrogel (gelatin, sodium alginate, etc.) through microfluidic or emulsification methods, and then obtains the fixed shape by illumination or crosslinking agent coordination (as shown in Figure 2A). The drug can be mixed into the solution during molding or loaded through the pores on its surface after the preparation of the MNMs.
(2) Exogenously actuatable MNMs can execute tasks in diverse environments by harnessing external stimuli or energy sources to drive their motions and operations (Leshansky et al., 2016). The exogenously actuatable MNMs are prepared by assembling field-responsive components (such as magnetic layers) inside or on the surface of the robot, and then coating the drug through special functional groups or functional layers (as shown in Figure 2B). These robots demonstrate excellent actuation performance and high dexterity, yet their biocompatibility is comparatively limited, and they rely on external energy sources (Wang et al., 2021b).
(3) Bio-based MNMs, including DNA nanorobots (Douglas et al., 2012), are fabricated using biomaterials and biomolecules and present good self-driving performance *in vivo* by using biomass or microorganism as main structure (Bao et al., 2018). For example, as shown in Figure 2C, a biocompatible asymmetric polymer vesicles achieved attractive chemotaxis driven by encapsulating glucose oxidase alone or in combination with catalase. These robots find applications within living organisms (Oral and Pumera, 2023). They possess desirable attributes such as biocompatibility, molecular recognition, and self-assembly capabilities, making them highly programmable. However, their actuation efficiency are relatively limited (Jiang et al., 2022).

The characteristics and fields of application of MNMs are intricately linked to their chemical composition and physical properties. These robotic systems hold immense promise in various domains, including medicine, nanotechnology, and bioengineering, presenting novel opportunities for problem-solving and achieving precise manipulation.

## 3 Classifications based on propulsion mechanisms

### 3.1 Chemically propelled MNMs

**FIGURE 3:**
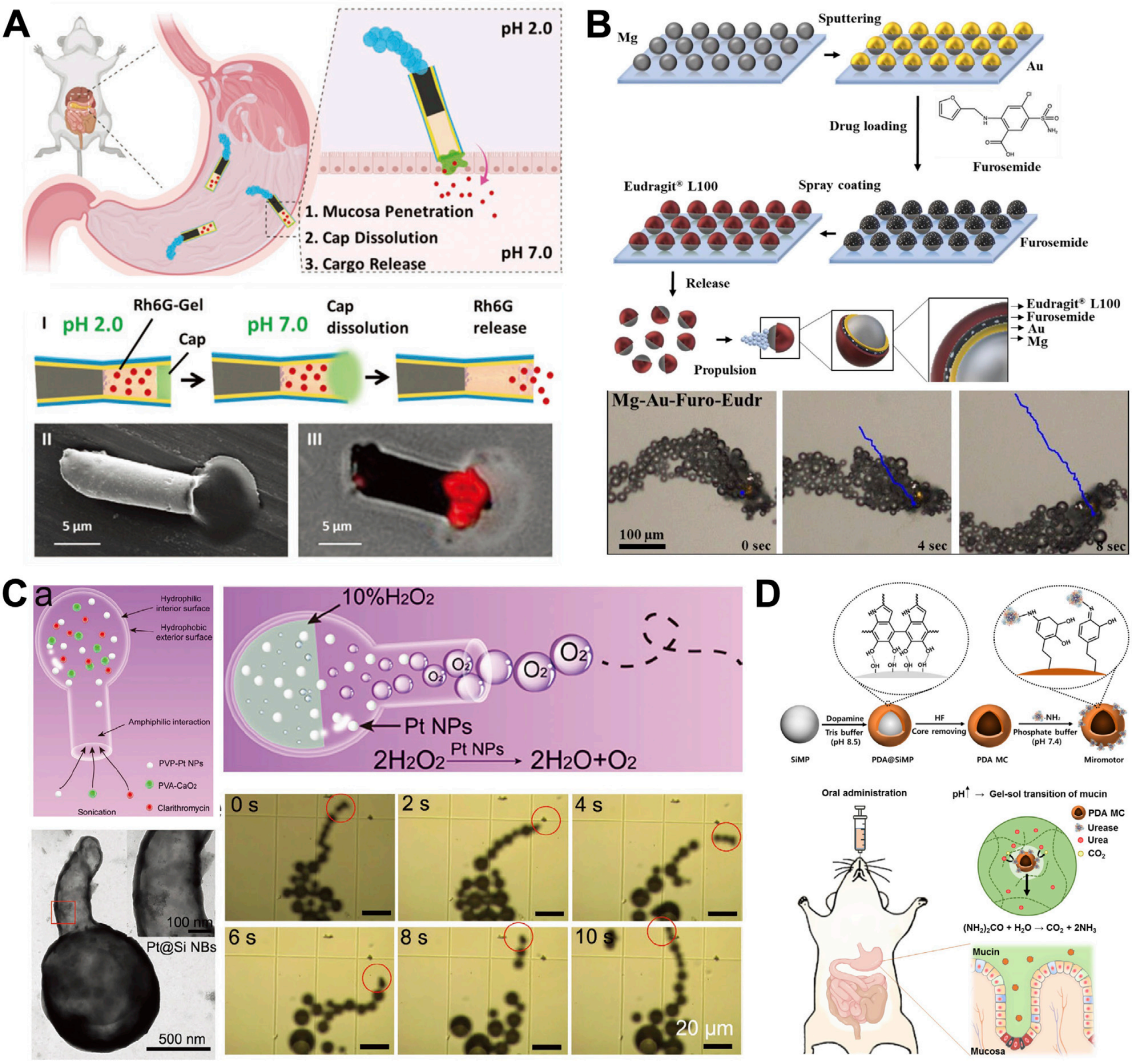
Chemically propelled MNMs. **(A)** Multicompartment tubular Zinc (Zn)-Based MNMs (Esteban Fernández de Ávila et al., 2020). **(B)** Self-propelled Janus Mg-Based MNMs (Maric et al., 2022). **(C)** Gastric acid powered MNMs (Wu et al., 2021). **(D)** Bioinspired urease-powered MNMs (Choi et al., 2022). **(A)** Copyright ^©^ 2020 WILEY‐VCH Verlag GmbH and Co. KGaA, Weinheim **(B)** Copyright ^©^ 2022 Maric T, Atladóttir S, Thamdrup LHE, Ilchenko O, Ghavami M, Boisen A. Published by Elsevier Ltd. **(C)** Copyright ^©^ 2021 Wiley‐VCH GmbH **(D)** Copyright ^©^ 2022 Bioactive Materials.

Chemically propelled MNMs are capable of harnessing specific chemicals in a given environment to generate propulsive forces through catalytic or spontaneous reactions (Huckaby and Lai, 2018). These MNMs include zinc (Zn)-based, magnesium (Mg)-based, CaO_2_/Pt NPs powered and enzyme-powered MNMs (Zhang S. et al., 2023). For chemically propelled MNMs, the actuation energy comes from chemical reactions, and the required energy to drive MNMs is correlated with the energy generated by chemical reactions. Each type of MNMs has its own characteristics and advantages (Figure 3). Metal-based chemically propelled MNMs can react with gastric acid, not only neutralizing it but also using natural biological resources like gastric acid to power metal-based motors, enhancing the retention and functionality of robots in the body (Li et al., 2017b). Although they are widely used, their biocompatibility and potential tissue damage remain concerns. On the other hand, enzyme-driven MNMs can tackle toxicity challenges in biomedical applications; however, the complex biological environment complicates their use, particularly regarding enzyme activity stability and mobility (Yuan et al., 2021).

#### 3.1.1 Zinc (Zn)-Based MNMs

In an acidic environment, Zn undergoes a spontaneous reaction with gastric acid protons, resulting in rapid autonomous directional thrust (Esteban Fernández de Ávila et al., 2020). Figure 3A describes this directed movement is facilitated by the creation of a galvanic cell between the Zn and the sputtered gold (Au) contact. The Zn body dissolves, autonomously releases the enclosed payloads and eventually disassembles the motors. The motors keep running until they are almost completely dissolved, ensuring that no toxic compounds are left behind (Sattayasamitsathit et al., 2014). This appealing functionality renders these Zn-based MNMs suitable for mobility and operation within the challenging stomach environment, thereby paving the way for the initial *in vivo* deployment of MNMs in living animals.

The first *in vivo* study of Zn-based (PEDOT/Zn) MNMs in a live mouse model demonstrated that acid-driven propulsion in the stomach effectively enhanced the binding and retention of the motors and their payload on the gastric wall (Gao et al., 2015). This *in vivo* evaluation of PEDOT/Zn MNMs assesses the distribution, retention, cargo delivery, and acute toxicity profile of synthetic motors in the mouse stomach through oral administration. The notable advantage was the biodegradation of PEDOT/Zn MNMs upon fulfilling their function. Clinical trials demonstrated that PEDOT/Zn tubular microrobots do not produce any toxic byproducts during their swimming, cargo delivery, and dissolution in gastric acid.

Lin et al. presented a microrod comprising a Zn core and a positively charged poly (3,4-ethylenedioxythiophene) (PEDOT^+^) shell for adsorbing anionic model drugs, sulforhodamine B (SRB^−^) (Cui et al., 2019). The MNMs can convey therapeutics to targeted sites and to actuate galvanically localized payload release, in response to the local pH. In the acidic gastric environment, the predominant reduction of H^+^ to H_2_ bubbles propels the delivery device as a micromotor. Galvanic payload release is facilitated by PEDOT + reduction, which predominates in the neutral stomach mucus layer.

A recent study team created a unique multi-compartment Zn-based MNMs that uses a pH-response gut cap to protect the cargo loading gelatin section and a rear end zinc-propellant engine (Esteban Fernández de Ávila et al., 2020). The MNMs propels due to the catalytic reaction between Zn and gastric juice, moving towards the stomach wall. The front end penetrates the stomach tissue, the enteric polymeric cap dissolves, and the therapeutic cargo is released. Results show that this multicompartment robot is more effective than the previously designed monocompartment, with no observed toxic residue. The improved distribution and retention of the multicompartment microrobots may stem from their innovative design. For monocompartment MNMs, the cargo is unprotected, allowing for gradual leakage. In contrast, multicompartment MNMs are less likely to experience leakage in acidic environments. The unique capabilities reported for Zn-based MNMs provide broad prospects for the gastric-targeted drug delivery.

#### 3.1.2 Magnesium (Mg)-Based MNMs

In addition to Zn-based MNMs, Mg-based MNMs are also frequently employed for gastric drug delivery. Mg-based MNMs operate on a principle similar to Zn-based MNMs. They can utilize the surrounding aqueous medium as a fuel to generate H_2_ bubbles, facilitating their movement (Děkanovský et al., 2022).

The research reported an enteric-coated MNM consisting of a Mg-based motor body with an enteric polymer coating (Li et al., 2016). The coating, which is soluble in neutral or alkaline environments but stable in acidic circumstances, guarantees accurate location in the GI system, while the magnesium body promotes self-propulsion in intestinal fluid. The enteric coating serves as protection against the acidic gastric fluid environment (pH 1–3), yet dissolves in intestinal fluid (pH 6–7), unveiling the motors to their fuel and initiating movement. Furthermore, by altering the intestinal coating’s thickness, one may control how long it takes for the polymer layer to dissolve, which in turn controls how far the motor must go down the GI tract before propulsion begins. The characteristics and capabilities of the created enteric Mg micromotors are assessed using a mouse model. *In vivo* findings indicate that these motors can successfully navigate through gastric fluid and precisely trigger activation in the GI tract without inducing noticeable acute toxicity.

A multifunctional Janus Mg-based MNM that can swim, neutralize acids, load, transport, and release payloads in response to pH variations was also developed by the research team (Li et al., 2017b). By utilizing acid as a fuel source, these synthetic motors rapidly consume protons while moving within the stomach, effectively raising the gastric pH to a neutral level in less than 20 min after application. Importantly, the autonomous neutralization of stomach fluid induced by the motors triggers the release of payloads from the pH-sensitive polymer coating. The MNMs dynamically alter the local environment without inhibiting the function of proton pumps. This approach minimally interferes with stomach function and eliminates potential adverse effects associated with traditional proton pump inhibitors (PPIs). The MNMs, composed of biocompatible materials without inherent biological activities, are safe and do not cause acute toxicity. In comparison to conventional pH-responsive nanocarriers that passively respond to the local environment, these MNMs actively modify their surroundings to achieve desired conditions for triggered payload release.

A study shown in Figure 3B delved into the feasibility of delivering a small-molecule drug under specific pH conditions utilizing MNMs, with furosemide serving as proof-of-concept drug (Maric et al., 2022). They developed self-propelled Mg-Au MNMs, loaded them with furosemide and coated them with pH-sensitive polymer (Eudragit^®^ L100) using ultrasonic spray coating technique.

Consequently, the use of Mg-based MNMs, which have the combined ability to neutralize acids and release payload in response to changes in pH, offers a unique and extremely promising platform for drug delivery in the treatment of a variety of gastrointestinal disorders.

#### 3.1.3 CaO_2_/Pt NPs powered MNMs

As a desirable fuel, hydrogen peroxide (H_2_O_2_) has found extensive applications. Certain metals, such as platinum (Pt), manganese (Mn), and others, can catalyze the decomposition of hydrogen peroxide into water and oxygen, thereby creating propulsive forces (Naeem et al., 2021).

Han et al. introduced a kind of hollow silica particle whose shape is a hollow bottle with a hydrophobic outer surface and a hydrophilic inner (called silica nanobottles, Si NBs) (Wu et al., 2021). As described in Figure 3C, the study’s Si-based nanomotor loads Pt NPs, nano CaO_2_, and clarithromycin (CLA) into a large cavity via amphiphilic interaction, resulting in a high drug loading capacity. Once delivered to the stomach cavity, the CaO_2_ component can transiently and physically alter the local acidic environment by rapidly consuming protons as it progresses in the stomach. Pt NPs catalytically decompose the product H_2_O_2_ into a substantial quantity of oxygen (O_2_). The concentration gradient of O_2_ bubbles locally expels them from the nanobottles through a narrow opening, propelling the nanobottles forward to achieve optimal release and enhance prodrug efficacy. Experiments in animal models show that this process effectively elevates the pH in the stomach to a neutral level, addressing the issue of chronic toxicity associated with prolonged use of PPI.

#### 3.1.4 Enzyme-powered MNMs

While Mg- and Zn-based MNMs have shown success in treating GI diseases, there remain challenges, particularly in terms of their biodegradability and catalytic reactions (Děkanovský et al., 2022). The residues that are difficult to degrade are the knotty problems. In addition, the propulsion of Mg/Zn-based MNMs through explosive and aggressive redox reactions of metals may lead to tissue damage when penetrating the GI tract. Therefore, MNMs powered by biological enzymes have gained increasing popularity in recent years.

Choi et al. (2022) developed bioinspired urease-powered polydopamine (PDA) micromotor as a biomimetic system for active oral drug delivery in the stomach (as shown in Figure 3D). The micromotor used urea in stomach as a bioavailable power source for the propulsion of synthetic motors. Under acidic conditions, the immobilized urease efficiently converts urea to ammonia, a process comparable to that in neutral conditions due to the increase in surrounding pH during propulsion. This demonstrates the feasibility of micromotors for *in vivo* stomach applications. Next, the micromotors were evaluated *in vivo* for improved penetration into the stomach wall and extended retention in the stomach. Furthermore, after a 3-day period, histological analysis was conducted to verify the safety and complete clearance of micromotors from the stomach. The results showed that the microrobots disappeared after 3 days, with no observed GI toxicity. The research confirmed the feasibility of urease-powered micromotors for the applications in the stomach (Choi et al., 2022).

### 3.2 External-field propelled MNMs

**FIGURE 4:**
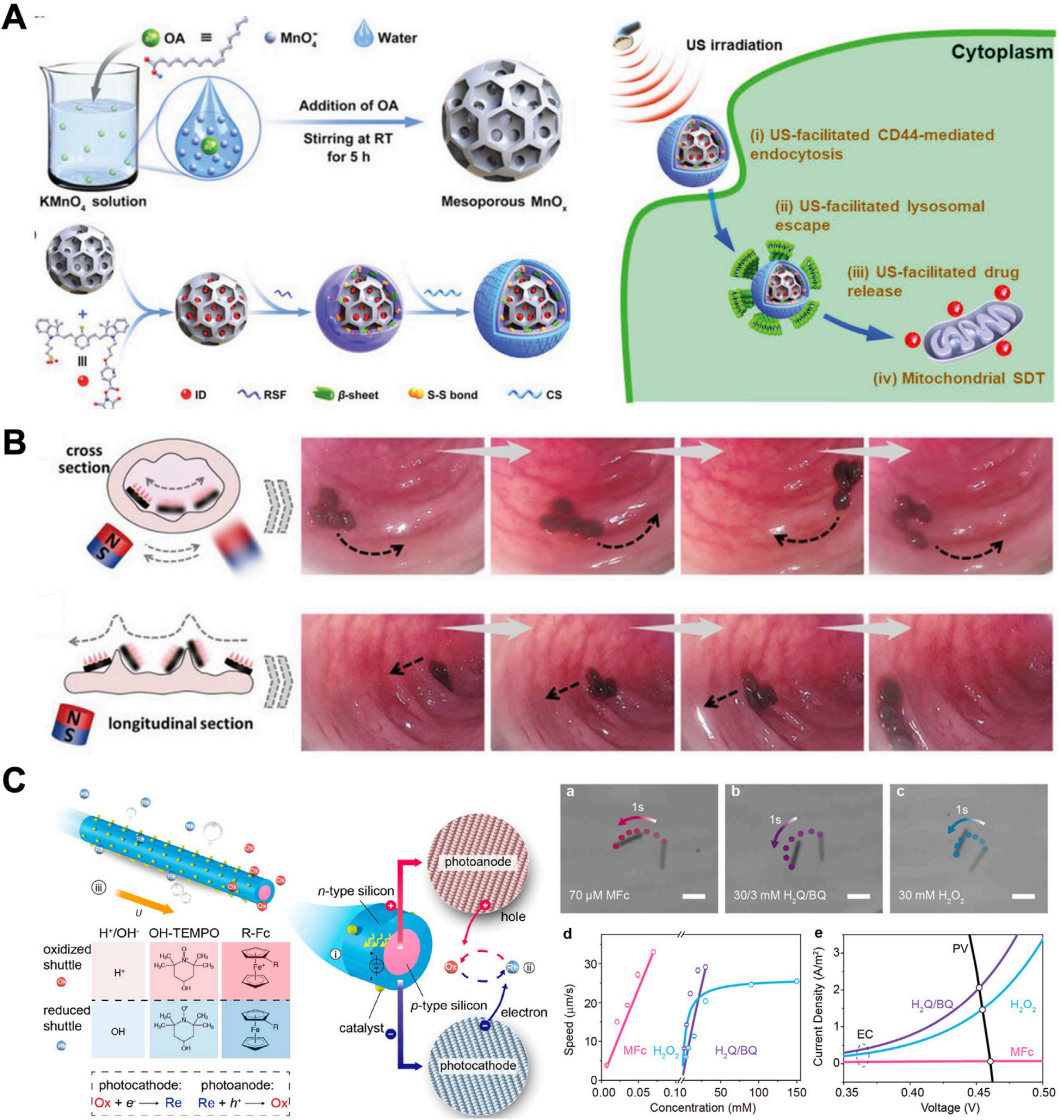
External-field propelled MNMs. **(A)** hydrogen peroxide (H_2_O_2_)/ultrasound (US)-driven mesoporous manganese oxide (MnOx)-based nanomotors (Cao et al., 2022). **(B)** Magneto-responsive microneedle robots (Zhang X. et al., 2021). **(C)** Highly efficient light-driven microswimmer (Wang J. et al., 2020). **(A)** Copyright ^©^ 2022 Wiley‐VCH GmbH **(B)** Copyright ^©^ 2021 Wiley‐VCH GmbH **(C)** Copyright Reprinted (adapted) with permission from {Rational Design of Reversible Redox Shuttle for Highly Efficient Light-Driven Microswimmer}. Copyright {2020} American Chemical Society.

While chemically propelled MNMs demonstrate effective propulsion, their utility is constrained by fuel scarcity and specific chemical reaction requirements (Luo et al., 2018a). External field-propelled MNMs (Figure 4) offer an appealing solution for practical applications, as they generally do not require external fuels, ensuring biocompatibility and sustainability (Xu T. et al., 2017). The category of externally driven MNMs includes devices powered by light, magnetic fields, or ultrasonic waves (Zhang S. et al., 2023). Unlike chemically propelled counterparts, these MNMs offer greater flexibility in controlling their motion, aligning more closely with the demands of active drug delivery.

#### 3.2.1 Ultrasound-driven MNMs

Serving as a stable external physical field, ultrasound (US) has a number of benefits, such as the cavitation effect, high tissue penetration efficiency, and non-invasiveness (Ou et al., 2020). US has demonstrated its high safety and efficiency as a power source capable of generating propulsive forces for MNMs (Ozcelik et al., 2018). Due to their distinctive features of biocompatibility and dependability, MNMs powered by US exhibit significant promise for a variety of biomedical applications (Wang and Gao, 2012).

Acoustically propelled MNMs employ diverse actuation mechanisms (Lu et al., 2019). Mallouk et al. (2018) initially described a self-acoustophoresis mechanism for rigid metallic rod-type micromotors, propelled in the MHz US frequency range (Wang et al., 2012). The levitation force generated by US propagation—generally regarded as the primary acoustic radiation force perpendicular to the substrate—caused the microrods to aggregate at the nodal plane during this phase. Additionally, the concave end of the microrods could create asymmetrical gradients of acoustic pressure, leading to self-phoresis of the micromotors. Thus, microrods suspended in a liquid might be driven to demonstrate in-plane rotation, alignment, and dynamic self-assembly in the levitation plane of the solution by changing the frequency-related vibration mode of the acoustic field. Kagan et al. (2012) introduced an alternative approach that employed US-induced vaporization to propel a metallic tubular micromotor. A metallic microtube was designed with a tapered conical structure, and its interior was filled with a perfluorocarbon (PFC) emulsion. Upon exposure to short US pulses, the tubular micromotor would initiate a “bullet-like” projectile motion through the vaporization of the acoustic droplets. Another form of acoustic activation for nanomotors involved oscillation-induced streaming. Ahmed et al. devised an acoustic microswimmer comprising a rectangular polymer body with one or more conical indentations, capable of spontaneously capturing microbubbles in a liquid environment (Ahmed et al., 2015).

In GI tract, H_2_O_2_/US-driven mesoporous manganese oxide (MnOx)-based nanomotors shown in Figure 4A are constructed by loading mitochondrial sonosensitizers into their mesoporous channels and orderly dual-functionalizing their surface with silk fibroin and chondroitin sulfate (Cao et al., 2022). Their movement is primarily dependent on the constant provision of chemical fuel (H_2_O_2_) and the effective tissue penetration capability of US. US-propelled MNMs exhibit high safety compared to those chemical propulsion methods, albeit with a limitation in directional control of their locomotion. US possesses the ability to penetrate biological tissues deeply with minimal adverse effects on biological systems, suggesting its potential for *in vivo* active drug delivery in GI tract.

#### 3.2.2 Magnetically driven MNMs

Magnetically driven MNMs address most disadvantages presented by others propulsion principles, and have the advantages such as fuel free, remote maneuverable, reconfigurable, programmable and multi-purpose (Li et al., 2023; Zhou et al., 2021; Yang, 2020). Magnetically propelled MNMs are made from magnetic materials, such as Ni and Fe_3_O_4_ (Erb et al., 2016). An external magnetic field serves as both a power source and guidance mechanism for MNMs, enabling controlled movement in specific directions or positions (Wang et al., 2023; Yu et al., 2022; Yu et al., 2024). For magnetically driven MNMs, the required energy to drive MNMs is mainly used to generate the external magnetic field, and the required energy to drive MNMs is approximately equal to the energy consumed by the magnetic field generating device. The hysteresis loss is the energy consumed by magnetic materials due to hysteresis during magnetization. The hysteresis loss of a magnetic material is proportional to the area of its hysteresis loop. Due to the small size of MNM, the magnetic materials used are paramagnetic, and the hysteresis loop area of paramagnetic materials is small. The hysteresis loss produced has little effect on the movement magnetically driven MNMs.

Viscosity is a property of a fluid that describes how easy it is to flow, and the magnetic field is generated by external magnetic field generating device. In general, the viscosity of the fluid does not affect the generation and strength of the magnetic field.

Due to the small size of MNMs, the Reynolds number is much less than one. At this time, the flow bulk state changes from turbulent flow to creep flow state, which is called Stokes flow. For a spherical MNMs with radius r in Stokes flow with viscosity η . The resistance exerted by the surrounding fluid will act as a hindrance along the direction of its motion velocity v . The drag force F of the fluid can be expressed as follows:

F=−6πηr·v

When the MNMs is rotated with an angular velocity ω . The drag moment T of the fluid can be expressed as follows:

$$T=-8\pi \eta r^{3}\cdot \omega$$

While viscosity does not affect the generation or strength of the magnetic field, it significantly influences the actuation of MNMs by imposing drag forces and moments.

**FIGURE 5:**
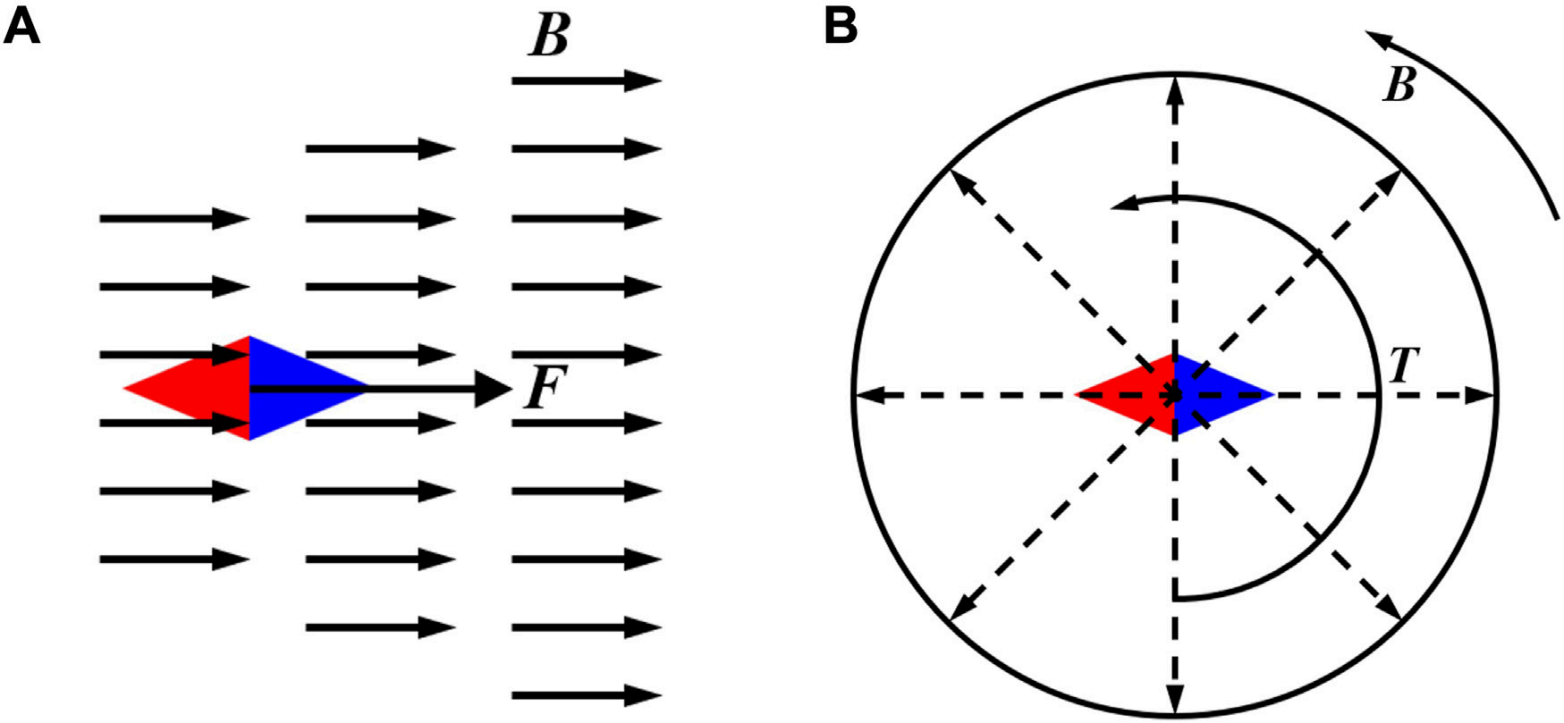
Magnetically driven MNMs in the external magnetic field. **(A)** In a gradient magnetic field. **(B)** In a rotating magnetic field.

Magnetically driven MNMs are subjected to different driving forces depending on the type of external magnetic field. When in a gradient magnetic field, the magnetic force on the magnetically driven MNMs is in the same direction as the magnetic induction gradient of the external magnetic field, and its magnitude is proportional to the magnitude of the magnetic induction gradient (Figure 5A). When the magnetic moments of the magnetically driven MNMs in the magnetic fields are not parallel to the external magnetic field, the MNMs will be rotated by the magnetic moment until the direction of the magnetic dipole is in the same direction as the external magnetic field (Figure 5B).

Zhou et al. (2019b) reported on a microdevice that consists of a poly (aspartic acid) (PASP) microtube, a thin Fe intermediate layer, and a core of Zn. This device can utilize gastric acid as a propellant. Once doxorubicin is adsorbed onto a PASP surface, the MNM becomes capable of transporting drugs, magnetically homing in on targets, penetrating the gastric mucus gel layer, and enhancing drug retention in the stomach without causing noticeable toxicity. All materials used in the MNMs are biocompatible and biodegradable, readily decomposable by gastric acid or proteases in the digestive tract. The incorporation of poly (amino acid) s in these MNMs expands their potential biomedical applications.

With their reputation for penetration, minimum invasiveness, and user-friendliness, microneedles have been widely used in drug delivery (Zhang S. et al., 2023). It is conceivable that the combination of drug-loaded microneedles and magnetically responsive MNMs could create an excellent oral delivery system for macromolecular drugs. Zhang X. et al. (2021) developed the magneto-responsive microneedle robots with the mentioned features using a multistage 3D fabrication strategy inspired by Lego brick stacking. Such microneedle robots consisted of three components, including the magnetic substrate, the separable connection, and the tips. Encasing these microneedle robots within a standard enteric capsule ensured stability in gastric juices and subsequent release in the small intestine (as shown in Figure 4B). Utilizing magnetic polarities in their substrate, the microneedle robot tips could orient themselves towards a specific magnetic direction. Consequently, upon release, the microneedle robots could navigate through small intestinal barriers and insert into the tissue with magnetic assistance. The separable connection would degrade, leaving the tips within the small intestinal tissue for protein delivery, while the magnetic substrate could be excreted.

Magnetically actuated propellers (MAPs) have been demonstrated to be highly suitable for various microfluidic and biotechnology applications (Mandal et al., 2018). Mandal et al., (2018) explored an alternative magnetic propulsion mechanism, utilizing oscillating magnetic fields to drive the MAPs. This approach induces motility in a back-and-forth motion, without specifying directionality, making the nanomotors effectively self-propelled entities with zero-force and zero-torque. Compared to their passive counterparts, the MAPs exhibit increased diffusivity, and their motion can be adjusted by modifying the external magnetic drive. This highlights the suitability of MAPs as model active particles.

In summary, magnetically driven MNMs possess the aforementioned advantages and hold broad prospects for drug delivery in the GI tract. However, the widespread adoption of magnetically propelled Micro/Nanomotors (MNMs) in the medical field is hindered by the necessity for specialized magnetic field generation equipment and intricate imaging instruments (Zhang S. et al., 2023).

#### 3.2.3 Light-driven MNMs

As a renewable energy source, light can be precisely controlled by adjusting parameters such as intensity, frequency, polarization ratio, and propagation direction (Liu C. et al., 2022). This enables reversible selection of the “on/off” mode of light, achieving non-invasive high spatial and temporal resolution for remote wireless control. There are three main physical driving modes for light-powered Janus MNMs, optical forces and torques, photothermal heating, and photocatalytic effects (Liu C. et al., 2022). The essence of optical tweezers is an expression of optical force generating from the energy and momentum exchange between light and particles. The photothermal effect is a phenomenon where a substance absorbs light energy and swiftly converts it into heat through electron–phonon coupling, leading to an increase in the temperature of the substance and its surrounding environment. Photocatalysis refers to the light-facilitated chemical reaction.

MNMs propelled by light are composed of at least one photoactive material, including photocatalytic materials, photochromic materials, photothermal materials, and others (Xu L. et al., 2017). Photocatalytic materials are the most widely explored for the fabrication of light-driven MNMs. For example, as shown in Figure 4C, Wang et al. (2012) developed a group of redoxactive but diffusion-sluggish ferrocene derivatives, enabling a record speed of ∼500 μm/s (∼100 body length per second) for self-electrophoretic light-driven micro/nanomotor at a biocompatible fuel concentration as low as 70 μM(70). Materials such as TiO_2_, Pt, AgCl, and others exhibiting photocatalytic activity are employed in the fabrication of light-driven MNMs (Zhou et al., 2020). Chen et al. (2017) constructed asymmetrical surface chemical reactions on the isotropic semiconductor particles by taking advantage of the limited penetration depth of light in semiconductor materials, which induced concentration gradients of photocatalytic products to propel the MNMs. Light-controlled isotropic TiO_2_ microrobots have the capability to produce non-electrolytic O_2_ molecules at the illuminated hemispherical surface through the photocatalytic breakdown of H_2_O_2_. The resulting osmotic O_2_ gradient induces a targeted local fluid flow, propelling the TiO_2_ microrobots towards areas with lower O_2_ concentration.

In addition to photocatalytic materials, photochromic materials have the ability to undergo reversible transformation between two isomers under light stimulus. This property enables changes in physical and chemical properties, including wettability, solubility, surface free energy, and liquid crystal alignment (Kay et al., 2007). Abid et al., (2011) developed azobenzene-coated polymer nanoparticles as light-driven MNMs. Azobenzene-coated polymer nanoparticles in the 16-nm-diameter range act as phototriggered nanomotors combining photo to kinetic energy conversion with optical control through light intensity gradients. Particle motion analysis reveals that photoisomerization fulfils a crucial role in transport, providing a measured driving force that is 3 to 4 orders of magnitude higher than optical forces.

Moreover, photothermal materials capable of generating photothermal effects under light irradiation, can be employed in the fabrication of light-driven MNMs. Lin et al. (2017) developed near-infrared light (NIR) propelled MNMs through integrating plasmonic gold nanoshells into nanoparticles or layer-by-layer assemblies in an asymmetric manner. Wang et al. (2012) have presented a biological chemotaxis-guided self-thermophoretic nanoplatform (BCTN) that facilitates precise intestinal positioning and autonomous mucus penetration (Lin et al., 2017). The nanoplatform incorporates mesoporous silicon with extraordinary drug-loading capacity as a matrix, partially coated with platinum to establish an asymmetric, autonomously movable nanoplatform. BCTN asymmetrically absorbs the NIR laser and autonomously penetrates the dense and viscous mucus barrier by self-thermophoretic propulsion force. In addition to supplying propulsive energy, NIR light possesses the capability for optical imaging, enabling the monitoring of the *in vivo* movement of MNMs (Pansare et al., 2012).

However, while light-driven MNMs have found extensive applications in bioanalytical fields, challenges persist for their broader clinical adoption (Liu C. et al., 2022). The primary concern revolves around the biosafety of MNMs. Opting for biodegradable and biocompatible materials in their construction is essential for favorable bioapplications. Moreover, a comprehensive biosafety assessment of MNMs is imperative, covering aspects such as immune response, biofouling, stability, biodistribution, and *in vivo* degradability. The toxicity associated with the commonly used fuel, H_2_O_2_, poses a significant limitation to the bioanalytical utility of MNMs. And the ideal medium, pure water, may not efficiently propel MNMs. Thus, obtaining highly efficient MNMs requires meticulous MNM design and fabrication that optimizes light absorption and catalysis capabilities.

### 3.3 Biohybrid MNMs

**FIGURE 6:**
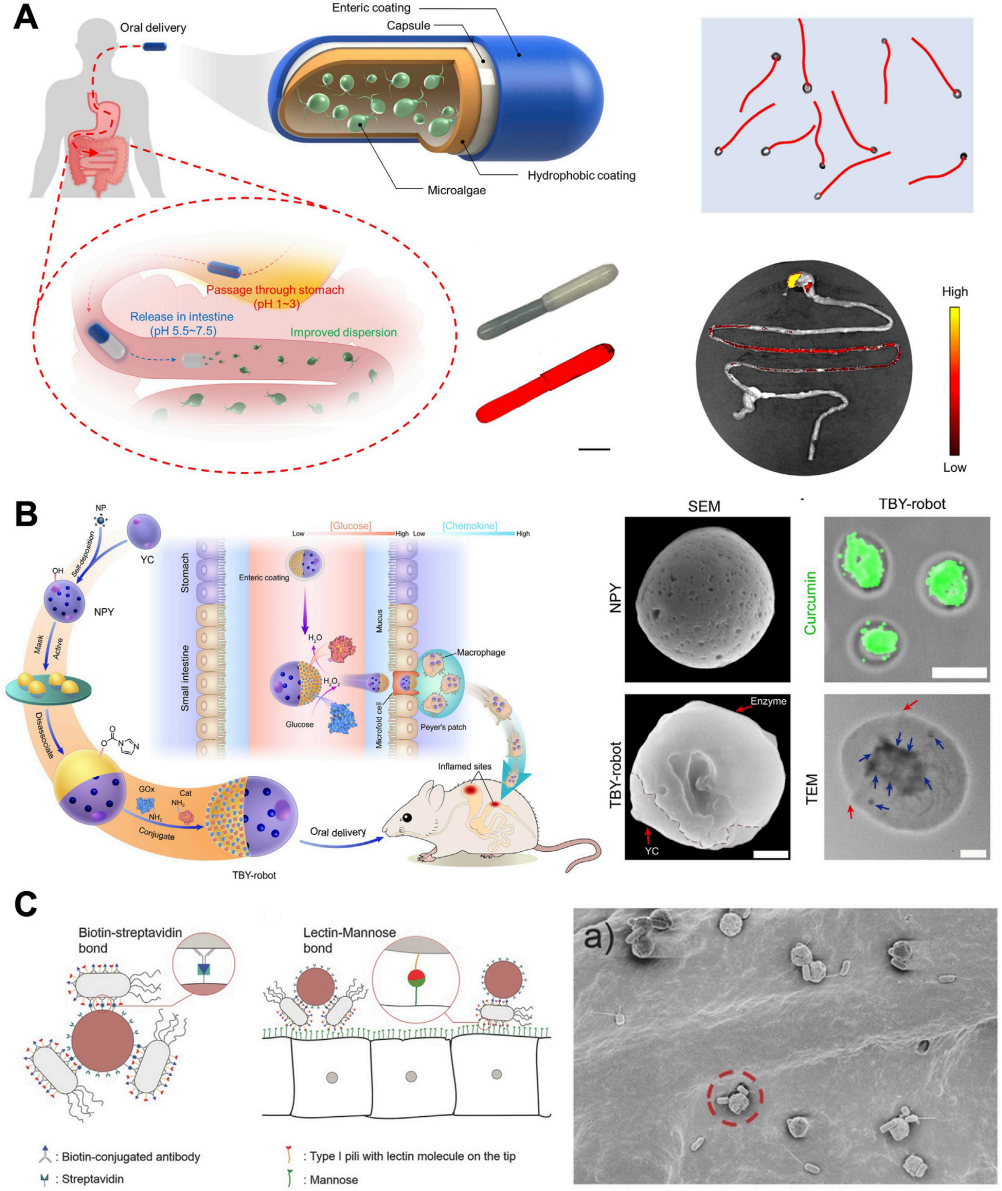
Biohybrid MNMs. **(A)** Efficient algaebased motor platform (Zhang et al., 2022b). **(B)** Twin-bioengine self-adaptive micro/nanorobots including enzyme actuation and macrophage relay (Zhang B. et al., 2023). **(C)** Bioadhesive bacterial microswimmers (Mostaghaci et al., 2017). **(A)** Copyright ^©^ 2022, The American Association for the Advancement of Science **(B)** From (Twin-bioengine self-adaptive micro/nanorobots using enzyme actuation and macrophage relay for gastrointestinal inflammation therapy). ^©^ Zhang B, Pan H, Chen Z, Yin T, Zheng M, Cai L, some rights reserved; exclusive licensee AAAS. Distributed under a CC BY-NC 4.0 license http://creativecommons.org/licenses/by-nc/4.0/. Reprinted with permission from AAAS. **(C)** Copyright ^©^ 2017 Mostaghaci B, Yasa O, Zhuang J, Sitti M. Published by WILEY‐VCH Verlag GmbH and Co. KGaA, Weinheim.

In contrast to artificially designed MNMs, motile microorganisms have undergone millions of years of evolution, exhibiting remarkable efficiency at microscales (Luo et al., 2018b; Zhang et al., 2023c). Through intricately integrated sensing and control pathways, they are able to respond to a variety of external stimuli, including as stresses, mechanical strain, and environmental chemicals. At the same time, they can turn chemical energy into mechanical effort (Carlsen and Sitti, 2014). Currently, bio-propelled MNMs (Figure 6) typically employ cells, microalgae, and bacteria for the treatment of GI diseases (Xu et al., 2022).

#### 3.3.1 Microalgae-based MNMs

Microalgae, abundant and renewable biological resources in nature, have garnered significant interest in biomedical applications (Zhang S. et al., 2023). Spirulina platensis (Sp) is a microorganism characterized by its naturally intact three-dimensional helical structure. It can be cultivated in large quantities and has already been commercialized as a nutritional supplement, showcasing its favorable feasibility and safety in biomedical applications (Yan et al., 2015). Yan et al. (2017) have presented biohybrid magnetic robots with versatile functionalities, incorporating specific structural and functional features from a biological matrix and an engineered coating. Utilizing a simple dip-coating process in magnetite (Fe_3_O_4_) suspensions, they fabricated helical microswimmers from Sp. These microswimmers are superparamagnetic and demonstrate robust navigation capabilities in diverse biofluids. The inherent properties of the microalgae enabled *in vivo* fluorescence imaging and remote diagnostic sensing without requiring any surface modification.

In addition to Sp, Chlamydomonas reinhardtii (*C. reinhardtii*) has good adaptability and motility. This microorganism can respond to visible light and exhibit phototaxis, demonstrating high swimming speeds in the range of 100–200 μm/s (Yasa et al., 2018). Their biocompatibility with healthy mammalian cells is demonstrated, and they are susceptible to surface changes for cargo bearing on their cell walls. They also move effectively in physiologically relevant environments and do not create known toxins. Yasa et al. (2018) have explored *C. reinhardtii* as the live component of biohybrid microrobots for the active delivery of therapeutics. They have presented a biohybrid algal microswimmer system with a high manufacturing yield achieved through molecular assembly of the nonliving component around the cell wall (Akolpoglu et al., 2020). The nonliving component comprises a conformal layer enveloping *C. reinhardtii* through the utilization of the natural biopolymer chitosan, facilitated by electrostatic interactions. In this process, the positively charged chitosan polymer binds to the negatively charged cell wall of *C. reinhardtii*. Acting as a binding agent, chitosan substantially enhances the subsequent attachment of nanoparticles. The thin coating of chitosan and nanoparticles has no detrimental impact on the motility and phototactic characteristics of the biohybrid microalgae. This study introduces a high-throughput manufacturing method for biohybrid microswimmers, laying the foundation for the advancement of a next-generation cargo delivery platform based on microalgae.

As described in Figure 6A, Zhang et al. (2022b) have reported on an efficient algae-based motor platform, which takes advantage of the fast and long-lasting swimming behavior of natural microalgae in intestinal fluid to prolong local retention within the GI tract. Compared to conventional magnesium-based micromotors, which have limits because of their brief propulsion lifetimes, the microalgae motors greatly improved the GI dispersion of the dye payload when given orally to live mice. Additionally, the microalgae motors improved the retention of a model chemotherapeutic payload in the GI tract when compared to a passive nanoparticle formulation.

While synthetic micromotors have proven useful for GI applications, their limited durability in acidic conditions restricts their practical deployment to short segments within the GI tract. Zhang et al. (2022c) have demonstrated an extremophile-based biohybrid micromotor capable of continuous and prolonged operation in extremely low pH environments. They relied on Chlamydomonas pitschmannii, an acidophilic alga isolated from an acid mine drainage (Dean et al., 2019). They additionally assessed the acidophilic biomotors’ functionality in gastric fluid (pH 1.5) and intestinal fluid (pH 6.5) while monitoring their *in vivo* biodistribution, aiming to determine their suitability for biomedical applications related to GI tract delivery. The results suggest that acidophilic algae-based biomotors with multifunctionality present unique benefits in comparison to conventional biohybrid platforms, demonstrating significant potential for biomedical applications related to the GI system.

In conclusion, biohybrid motor systems based on natural algae offer significant potential for oral drug delivery, contributing to the improvement of treatments for GI diseases. This innovative approach represents an appealing avenue for various biomedical applications.

#### 3.3.2 Bacteria-based MNMs

Bacteria utilize the biochemical energy within their membranes to propel themselves and respond to environmental gradients such as nutrients, oxygen, pH, or external fields. Consequently, they have been extensively employed as drivers for MNMs in oral drug delivery for GI diseases (Hosseinidoust et al., 2016). Stanton et al. (2016) introduced an innovative micromotor comprised of nonpathogenic *Escherichia coli* and a Janus particle. The metal-coated surface exhibited an affinity for adhering to bacteria, leaving an alternate surface for drug attachment. The iron in the Janus particle’s metal cap was used to determine the direction of motion using an external magnetic field, and *E. coli* contributed to the motion.

Mostaghaci et al. (2017) have proposed an approach to attach bacteriabots to certain types of epithelial cells (expressing mannose on the membrane), based on the affinity between lectin molecules on the tip of bacterial type I pili and mannose molecules on the epithelial cells (as shown in Figure 6C). By using lectin molecules, the bacteria were able to attach themselves to mannose-expressing surfaces and cells. In the context of bacteriabots, this ability also allowed the bacteria to anchor therapeutic drug-containing particles. Releasing the drug directly at the disease site can markedly enhance drug delivery efficiency and mitigate certain side effects. The utilization of this active bioadhesive approach holds great potential for future targeted drug delivery applications of bacteriabots.

Despite the appealing attributes of bacteria, several challenges of bacterial-based biohybrid MNMs must be addressed. Firstly, greater attention must be paid to safety concerns, particularly regarding the pathogenicity of bacteria. Additionally, it is important to take into account the activities of bacteria because certain of them may show decreased fitness when they come into touch with specific surfaces, which could affect the effectiveness of the motor systems (Xu et al., 2022). Researchers remain optimistic about the increasingly significant role that bacterial biohybrid micro/nanomotors will play in the field of MNMs.

#### 3.3.3 Other cell-based MNMs

Cell is the basic structural and functional unit of an organism (He et al., 2015). Materials derived from cells can effectively mitigate environmental pollution, reduce energy loss, and enhance safety measures (Elbagory et al., 2021). Zhang L. et al. (2023) have designed a self-driving yeast micro-/nanorobot (Cur@CaY-robot) via dual biomineralization and acid catalysis of calcium carbonate (CaCO_3_). The biomineralization process within yeast cells results in the formation of inner nano-CaCO_3_ (CaY) through cell respiration, providing nanoscaffolds for efficient encapsulation of curcumin (Cur). Simultaneously, the outer-CaCO_3_ crystals, formed outside yeast cells through uniaxial growth, serve as an asymmetric power source for self-propelled motility. The robotic system exhibited effective movement in gastric acid and successfully penetrated the dense gastric mucus, leading to a substantial enhancement in the accumulation of drug agents within the stomach wall tissue.

Macrophages, characterized by migration and chemotaxis capabilities, can traverse biological barriers and navigate toward inflamed sites guided by the concentration gradient of chemokines (Shi and Pamer, 2011). Zhang B. et al. (2023) have developed a twin-bioengine yeast micro/nanorobot (TBY-robot) with self-propelling and self-adaptive capabilities that can autonomously navigate to inflamed sites for GI inflammation therapy via enzyme-macrophage switching (EMS) (as shown in Figure 6B). Asymmetric TBY-robots successfully traversed the mucus barrier and significantly prolonged their retention in the intestine by employing a dual enzyme-driven propulsion system in response to an enteral glucose gradient. The TBY-robot then moved to Peyer’s patch, where the enzyme-driven engine changed to an *in situ* macrophage bioengine, allowing it to follow a chemokine gradient to inflamed areas. The self-adjusting TBY-robots present a secure and auspicious approach for precisely treating GI inflammation.

In addition, the use of microorganisms such as sperm, neutrophils, platelets, and red blood cells in MNMs design has been explored and promising results have been achieved (Chen et al., 2023). Nevertheless, numerous challenges remain. Firstly, the immune system’s response and removal processes will significantly impact the outcomes of micro/nanomotors. Moreover, ethical and moral considerations should be taken into account, even though they have not been extensively explored (Xu et al., 2022).

## 4 Applications of MNMs in GI disease

### 4.1 Regulation of gut microbiome

The intestinal barrier’s structural integrity, nutrition metabolism, host immunomodulation, and pathogen defense are all significantly impacted by the gut microbiome (Rinninella et al., 2019). Traditional oral probiotics face challenges such as poor bioavailability and limited efficacy in improving health outcomes (Suez et al., 2019). In recent years, fecal microbiota transplantation (FMT) has gradually gained attention. However, its poor compliance and the inevitable uncertainty regarding its components may lead to GI irritation, potential complications, and risks of severe or life-threatening infections (Blaser, 2019). Therefore, there is a strong desire for alternative intervention strategies that are easy to implement and address safety concerns for effective modulation of the gut microbiome.

**FIGURE 7:**
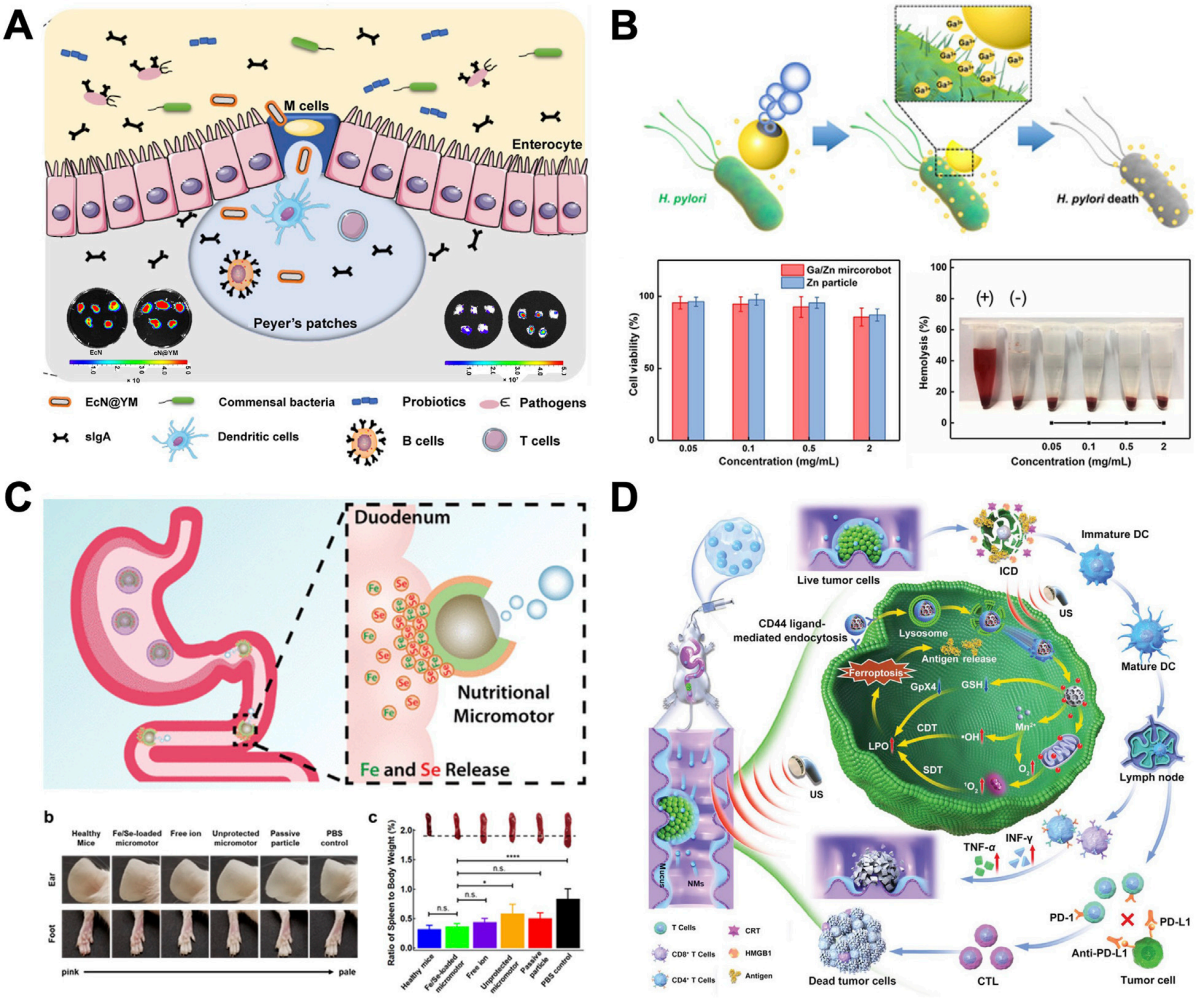
The application of MNMs in the GI tract. **(A)** Mucosal immunity–mediated modulation of the gut microbiome by oral delivery of probiotics into Peyer’s patches (Lin S. et al., 2021). **(B)** Bubble‐propelled janus gallium/zinc micromotors for active treatment of bacterial infection (Lin Z. et al., 2021). **(C)** Micromotors for active delivery of minerals toward the treatment of iron deficiency anemia (Karshalev et al., 2019). **(D)** Oral nanomotor-enabled mucus traverse and tumor penetration for targeted chemo-sono-immunotherapy against colon cancer (Cao et al., 2022). **(A)** From (Mucosal immunity–mediated modulation of the gut microbiome by oral delivery of probiotics into Peyer’s patches). ^©^ Lin S, Mukherjee S, Li J, Hou W, Pan C, Liu J, some rights reserved; exclusive licensee AAAS. Distributed under a CC BY-NC 4.0 license http://creativecommons.org/licenses/by-nc/4.0/. Reprinted with permission from AAAS. **(B)** Copyright ^©^ 2021 Wiley‐VCH GmbH, **(C)** Reprinted (adapted) with permission from {Micromotors for Active Delivery of Minerals toward the Treatment of Iron Deficiency Anemia}. Copyright {2019} American Chemical Society, **(D)** Copyright ^©^ 2022 Wiley‐VCH GmbH.

Lin et al. (2017) describe a bioinspired strategy of self-coating with biofilms (Wang X. et al., 2020). Employing clinical *Bacillus subtilis* as a representative probiotic bacterium, biofilm-coated probiotics exhibit significantly enhanced GI tract tolerance and mucoadhesion in both mice and swine. They also propose the elicitation of mucosal immunity to modulate the gut microbiota by oral delivery of living probiotics into Peyer’s patches (PPs) (Lin S. et al., 2021) (as shown in Figure 7A). Living probiotics are engineered for oral delivery to PPs, as antigen sampling through microfold cells (M cells) is the primary pathway for initiating mucosal immune responses. Beneficial bacteria are encapsulated within a yeast membrane (YM) using physical coextrusion through a porous membrane. Because of the presence of embedded β-glucan on YMs, after oral ingestion, coated bacteria can facilitate Dectin-1 receptor–mediated phagocytosis of M cells distributed in the intestinal epithelium. Administering camouflaged bacteria effectively alleviates gut barrier damage and reduces intestinal permeability in two murine models of intestinal barrier impairment. The study reveals how the composition and function of the gut microbiome can be preserved despite environmental challenges, and it suggests a novel platform for advancing oral therapeutics aimed at bacteria-mediated prevention and treatment.

### 4.2 Neutralization of gastric acid and treatment of *Helicobacter pylori* (*H. pylori*) infection

*Helicobacter pylori* infection plays a significant role in the development of gastroduodenal ulcer disease and gastric carcinoma (Fischbach and Malfertheiner, 2018). Typically, antibiotics are prescribed alongside proton pump inhibitors (PPIs) to treat *H. pylori* infection (Holtmann et al., 1999). PPIs help decrease gastric acid production, which can otherwise diminish the effectiveness of antibiotics. PPIs can irreversibly bind to proton pumps, thereby inhibiting acid secretion. However, long-term use may lead to risks such as nutrient deficiencies, increased risk of fractures, pneumonia, and intestinal infections (Sheen and Triadafilopoulos, 2011; Moayyedi and Leontiadis, 2012). Hence, it would be advantageous to devise an alternative treatment regimen that matches or surpasses the therapeutic effectiveness of current antibiotic therapies, while eliminating the need for PPIs.

Wang et al. (2012) represent the inaugural endeavor to utilize magnesium-based micromotors loaded with the antibiotic drug clarithromycin (CLR) for treating *H. pylori* infection in a murine model (de Ávila et al., 2017). With its inherent proton depletion capability, this motor-based therapy can navigate through the hostile gastric environment to exert antibacterial effects without necessitating the use of conventional PPIs. Unlike acid suppression via PPIs, Mg-based micromotors can dynamically modify the local acidic milieu by rapidly depleting protons while navigating through the stomach (Li et al., 2017b). When compared to inert drug carriers, the motion of drug-loaded magnesium micromotors in gastric fluids enables effective antibiotic delivery, leading to a significant decrease in the bacterial load in the mouse stomach with no observable toxicity.

He et al. (2015) report a bubble-propelled Janus gallium/zinc (Ga/Zn) micromotor with good biocompatibility and biodegradability for active target treatment of bacteria (Lin Z. et al., 2021). Figure 7B shows these biocompatible micromotors degrade completely in gastroenteric acid, releasing GaIII, which exhibits potent antibacterial properties. The movement improves the diffusion of GaIII and results in a significant increase of the antibacterial efficiency against *H. pylori*, compared with passive Ga microparticles. Such liquid metal micromotors, combining autonomous movement and biocompatibility, offer a promising avenue for actively targeting bacterial infections in gastroenteric environments.

### 4.3 Dynamic delivery of minerals

Inorganic minerals are crucial for regulating various metabolic pathways (Gupta and Gupta, 2014). Iron (Fe) deficiency is the most prevalent nutritional deficiency globally, carrying significant health and economic burdens (Zimmermann and Hurrell, 2007). The primary cause of Fe deficiency is often inadequate daily dietary intake. Approaches to replenish Fe typically involve dietary adjustments to incorporate Fe-rich foods, oral Fe supplementation, or intravenous Fe therapy (Armstrong, 2017). However, excess Fe can promote the growth of pathogenic bacteria in the intestines, displacing beneficial bacterial species, leading to inflammation, endotoxemia, and subsequent reduced nutrient absorption (Stelle et al., 2019). Selenium (Se) is a vital element in the enzyme glutathione peroxidase, playing a crucial role in antioxidant processes that safeguard cells from damage (Cueto, 2001). Se has a narrow range between effective (<40 μg/day) and toxic (>400 μg/day) levels, requiring careful attention to prevent inadvertent accumulation above the safety threshold during supplementation (Selinus et al., 2005). Therefore, it is evident that maintaining appropriate mineral levels in the body is crucial, as both deficiency and excess can have adverse effects on human health. Developing intelligent and dependable approaches to deliver multiple minerals, maximizing absorption and effectiveness while minimizing side effects, would be extremely advantageous.

Wang et al. (2012) present a study on the advancement of active mineral delivery systems employing Mg-based micromotors (Karshalev et al., 2019). As shown in Figure 7C, these micromotors can autonomously navigate through GI fluids, facilitating the dynamic transportation of minerals. The Fe and Se minerals were encapsulated within a chitosan layer and further shielded by an enteric pH-responsive polymer to inhibit premature mineral release in the stomach. The mineral-loaded micromotors were designed to be protected from the highly acidic stomach gastric fluid by an outer pH-responsive enteric coating, Eudragit^®^ L100-55, which is soluble only when pH ≥ 5.5. Blood levels of Fe and Se showed enhanced restoration compared to anemic control groups, while analyses of blood and organs showed no signs of toxicity after 30 days of treatment. These findings from *in vivo* experiments suggest that micromotors offer a promising approach for the targeted and active delivery of mineral supplements, especially for combating anemia.

### 4.4 Synergistic treatment of colorectal cancer (CRC)

CRC is the third most prevalent cancer worldwide (Garrett, 2019). While conventional therapies such as surgery, chemotherapy, and radiotherapy may temporarily inhibit the growth of primary colon tumors, they frequently prove ineffective in eradicating deeply embedded tumor cells and metastatic lesions (Li et al., 2022). The oral route offers advantages such as self-medication, good patient compliance, and direct delivery of therapeutic drugs to the colonic mucosa. However, the therapeutic efficacy of oral medications is hindered by various bottlenecks, including the mucous barrier, dense fibrotic stroma in colonic tumor tissue, and inefficient activation of anti-tumor immunity (Li et al., 2022).

To create a multifunctional drug delivery system, Cao et al. (2022) incorporated indocyanine green derivatives (IDs) with mitochondria-targeting properties into mesoporous MnOx, functionalized their surface with regenerated silk fibroin (RSF) and chitosan (CS), and then embedded the resulting nanomaterials (CS-ID@NMs) into a chitosan/alginate hydrogel (as shown in Figure 7D). The nanomaterials (NMs) could be selectively taken up by colon tumor cells via chitosan receptor (CD44)-mediated endocytosis. Upon exposure to triggers such as hydrogen ions, reactive oxygen species (ROS), glutathione (GSH), and US, the loaded IDs would be released in the cytoplasm. Consequently, tumor cells could be effectively eradicated through the combined action of chitosan-ID@NMs, which induce both manganese ion (Mn^2+^)-mediated chemodynamic therapy (CDT) and mitochondrial sonodynamic therapy (SDT). Additionally, this approach may activate adaptive immune responses. Furthermore, *in vivo* research has demonstrated that concurrent use of oral chitosan-ID@NMs and PD-L1 checkpoint inhibitors can successfully suppress the primary tumor and strengthen the systemic anti-tumor immune response, resulting in long-lasting immune memory effects and averting tumor recurrence. This oral nanoplatform could serve as a robust therapeutic system for achieving effective synergistic treatment of colon cancer and represents a promising nanoplatform for clinical translation.

## 5 Conclusion

The application of MNMs for targeted delivery in the GI tract holds tremendous potential. These MNMs can achieve precise drug delivery through oral administration, targeting diseases within the GI tract. They can overcome physiological barriers in the GI tract, such as mucosal layers and tumor tissues, enhancing the bioavailability and therapeutic efficacy of drugs. Furthermore, by releasing drugs in response to particular biological conditions or outside stimuli, these MNMs can accomplish regulated drug release, which improves therapeutic efficacy and minimizes side effects. By combining nanotechnology and principles of biology, targeted delivery systems and MNMs in the GI tract offer new possibilities for treating GI diseases and have broad clinical applications.

The research summarized in this article indicates that GI micro/nanorobots can autonomously propel and navigate in the complex environment of the GI tract, overcoming biochemical, mucosal, and epithelial barriers to actively and effectively achieve precise delivery of specific therapeutic drugs. However, the dynamic environment and physiological obstacles within the GI tract can affect the control of micro-robots’ motion trajectories, thereby compromising the precision of treatment and drug delivery. There are still many challenges that GI MNMs face in achieving clinical applications in the GI tract:

(1) The environmental barrier of the GI tract. The mucus secreted by the gastrointestinal tract has high viscosity and a certain acidity and alkalinity, which imposes high demands on the efficient movement of MNMs in this non-Newtonian fluid driven by external fields/fuels. Additionally, MNMs face significant challenges in their motion control due to the disturbances caused by peristalsis and fluid flow during navigation.
(2) Biological safety requirements. The internal application of MNMs in the GI tract not only imposes requirements for the biocompatibility and degradability of the MNMs’ materials but also needs to consider the potential local environmental impact caused by reactions between microrobots and biological fluids. This is particularly important to ensure that the original functions of the GI tract (such as digestion) are not compromised.
(3) Precise imaging. Current clinical imaging tools are limited by resolution, sensitivity, and imaging contrast, often only suitable for *in vivo* imaging of clusters of MNMs. Imaging of individual microrobots for precise surgery and real-time tracking during task execution in GI tract remains challenging.
(4) The actuation failure of MNMs. The actuation failure of MNMs may fall into two categories:
 • Resistance greater than actuation force. The resistance of MNMs exceeds the actuation force, and the resistance may come from various reasons, including the friction of the surrounding environment, adsorption forces with surrounding objects and et al. In this case, the MNMs will remain stationary or perform unstable movements, unable to carry out the intended work.
 • Disappearance of the actuation force. For chemically propelled MNMs when the fuel runs out, or for magnetically driven MNMs the external magnetic field generating device stops working. Due to the small size of MNMs, it can be regarded as its movement in Stokes flow. MNMs will stop moving quickly after the actuation force disappears.

Nonetheless, MNMs exhibit tremendous potential for precise therapies within the GI tract. Researchers in the field should continue designing and fabricating multifunctional microrobots, as well as developing integrated navigation systems that utilize specific imaging tools to track their trajectories.
